# Effects of Mild Blast Traumatic Brain Injury on Cognitive- and Addiction-Related Behaviors

**DOI:** 10.1038/s41598-018-28062-0

**Published:** 2018-07-02

**Authors:** Matthew J. Muelbl, Megan L. Slaker, Alok S. Shah, Natalie N. Nawarawong, Clayton H. Gerndt, Matthew D. Budde, Brian D. Stemper, Christopher M. Olsen

**Affiliations:** 1 Department of Pharmacology and Toxicology, Medical College of Wisconsin, 8701 Watertown Plank Rd., Milwaukee, WI 53226 USA; 2 Neuroscience Research Center, Medical College of Wisconsin, 8701 Watertown Plank Rd., Milwaukee, WI 53226 USA; 3 Department of Neurosurgery, Medical College of Wisconsin, 8701 Watertown Plank Rd., Milwaukee, WI 53226 USA; 4 Clement J. Zablocki Veterans Affairs Medical Center, 5000 W National Ave, Milwaukee, WI 53295 USA

## Abstract

Traumatic brain injury (TBI) commonly results in cognitive and psychiatric problems. Cognitive impairments occur in approximately 30% of patients suffering from mild TBI (mTBI), and correlational evidence from clinical studies indicates that substance abuse may be increased following mTBI. However, understanding the lasting cognitive and psychiatric problems stemming from mTBI is difficult in clinical settings where pre-injury assessment may not be possible or accurate. Therefore, we used a previously characterized blast model of mTBI (bTBI) to examine cognitive- and addiction-related outcomes. We previously demonstrated that this model leads to bilateral damage of the medial prefrontal cortex (mPFC), a region critical for cognitive function and addiction. Rats were exposed to bTBI and tested in operant learning tasks several weeks after injury. bTBI rats made more errors during acquisition of a cue discrimination task compared to sham treated rats. Surprisingly, we observed no differences between groups in set shifting and delayed matching to sample, tasks known to require the mPFC. Separate rats performed cocaine self-administration. No group differences were found in intake or extinction, and only subtle differences were observed in drug-primed reinstatement 3–4 months after injury. These findings indicate that bTBI impairs acquisition of a visual discrimination task and that bTBI does not significantly increase the ability of cocaine exposure to trigger drug seeking.

## Introduction

Recent estimates indicate that over two million Americans suffer a traumatic brain injury (TBI) each year^1^. Two of the most prevalent chronic outcomes of TBI are cognitive and psychiatric problems, and these are cited as the major contributor to long-term disability^2–4^. Substance abuse is one of the most common psychiatric problems following TBI, with estimated prevalence rates of 5–28%^5–8^ and relative risk of 1.8 for substance abuse at 13–24 months following injury^9^.

Among TBIs, approximately 75% are categorized as mild (mTBI)^10^. The American Congress of Rehabilitation Medicine has defined mild TBI as head trauma resulting in one of the following: loss of consciousness for less than 30 min, alteration of mental state for up to 24 h with a Glasgow coma score ≥13, or pre- or post-traumatic amnesia^11^. This definition roughly coincides with categorizations of mild head injury and concussion^12,13^. mTBI symptoms commonly resolve within a few weeks or months following injury, and many studies report 30% or fewer patients exhibit cognitive or behavioral problems that persist longer than three months^14–16^. However, a recent scoping review suggests that approximately 50% of mTBI patients may exhibit cognitive problems beyond three months^17^. Cognitive difficulties are one of the most commonly reported persistent symptoms in mTBI sufferers^14,17^. In a study of 122 mTBI patients, 26% reported cognitive impairment three months post-injury, and neuropsychological testing revealed impairment in 30% of patients^18^. Meta-analysis of 39 studies including over 2600 cases found that the overall effect of mTBI on cognitive function three months post-injury was low (d = 0.04), however the effect was much larger (d = 0.74) when analysis was restricted to clinic-based studies^19^. Among cognitive impairments reported, executive dysfunction is one of the most commonly reported persistent symptoms in mild and non-mild TBI^20,21^. Executive dysfunction can manifest in problems with adaptability, problem solving, and mental flexibility, which commonly disrupts interpersonal relationships and other aspects of quality of life^20^.

There is also evidence of increased substance abuse following mTBI, although interpretation is complicated by the fact that many studies either combine injuries of varying severity or do not report injury severity^9,22^. Nonetheless, compelling evidence comes from studies specifically studying mTBI. In a study of nearly two million discharged military personnel, the relative risk of discharge for alcoholism or drug use was 2.6 higher for those suffering from mTBI^23^. A longitudinal study of subjects who suffered an mTBI at five years of age or younger found an odds ratio of 3.6 for likelihood of substance abuse during adolescence, several years following the injury^24^.

Blast-related TBIs are commonly sustained in modern combat, urban terrorist attacks, and industrial accidents^25^. Blast is the leading cause of TBI in recent military conflicts; a 2010 report noted that 69% of TBIs occurring in operation enduring freedom/operation Iraqi freedom, now operation new dawn (OEF/OIF/OND), were from blast exposure^26^, and the estimated prevalence of blast-related TBI in OEF/OIF/OND personnel is approximately 20%^27–29^. Like non-blast mTBI, cognitive problems are among the most frequently self-reported enduring symptoms following blast mTBI^29,30^. Despite the high prevalence of self-reported cognitive problems following blast injury in military personnel, neuropsychological testing has not always shown mTBI-associated deficiencies, or that deficiencies may be confounded by comorbid Axis I psychopathologies such as PTSD or depression^31–34^. As with non-blast TBI (described above), there is evidence that executive dysfunction may be specifically altered in blast mTBI^35^.

Many factors complicate our understanding of cognitive and addiction-related sequelae of brain injury. In clinical studies, factors such as pre-injury cognitive performance or substance use history are often unclear^36^. Outcomes following injury are often self-reported, and cognitive function may not be rigorously tested following injury. On the other hand, biases such as the “nocebo effect” (outcomes associated with expectations of negative outcomes^37^), diagnosis threat (outcomes shaped by negative expectations associated with diagnosis^38^), and the potential for secondary gain from injury-related disability may overestimate rates of chronic impairment^39^. Furthermore, cognitive dysfunction and psychostimulant abuse are common co-morbidities^40,41^, and this relationship has been modeled in preclinical studies^42^. Preclinical studies can address many of these problems by controlling pre-injury factors and removing biases inherent in human studies.

Our blast model of mTBI (bTBI) has been demonstrated to produce cognitive and behavioral alterations several weeks following injury^43–45^. We have demonstrated that this injury model leads to bilateral alterations in the medial prefrontal cortex (mPFC), revealed by diffusion tensor imaging (DTI) and elevated glial fibrillary acidic protein (GFAP), a marker of astrogliosis^43^. Altered DTI outcomes (e.g., fractional anisotropy, mean diffusivity) have been extensively reported in the prefrontal cortex and associated white matter tracts in human mTBI^46–48^. Furthermore, lower fractional anisotropy values in prefrontal cortex have been correlated with greater executive dysfunction^46^. The rodent mPFC is an ortholog of the human medial and dorsolateral prefrontal cortices^49–51^, areas implicated in executive dysfunction and drug craving/seeking in both species^49,52–55^. We used sophisticated behavioral models to test the hypothesis that bTBI would disrupt performance in complex mPFC-dependent cognitive tasks and elevate drug seeking following cocaine self-administration.

## Materials and Methods

### Animals

Male Sprague Dawley rats (n = 54; Harlan) were used in this study. Rats arrived at the Zablocki Veterans Affairs Medical Center (ZAMC) at approximately 8 weeks of age and were initially housed in pairs. Rats were transferred from the ZAMC to the Medical College of Wisconsin (~5 miles away) three days after blast exposure or sham treatment. Upon arrival at the Medical College of Wisconsin, rats were singly housed for the remainder of the study. Food and water were provided ad libitum until 3 days prior to onset of cognitive testing, at which point food intake was restricted to 5.5 g of food per 100 g body weight per day until behavioral testing was complete. Rats were housed under reverse light cycle conditions (lights off 0730–1930), and experiments were performed during the dark period. Experimenters were blind to group assignment in all experiments. All experiments done in accordance with *The Guide for the Care and Use of Laboratory Animals (8*^*th*^ *ed.)* and were approved by the Institutional Animal Care and Use committee at the Medical College of Wisconsin.

### Blast Exposure

Rats were exposed to either a blast overpressure or sham conditions as described previously^43–45^. In brief, all rats were maintained under anesthesia with a continuous delivery of 1.5% isoflurane and had the torso shielded with a metal cylinder while the head was restrained to prevent rotational acceleration injury. Blast-exposed rats were placed 17 cm from the end of a custom shock tube (3.6 cm inner diameter, 3.0 m driven section, and 0.3 m driver section with mylar membrane between the driver and driven sections). Blast overpressure (450 kPa peak, 80 kPa*msec) was then produced by pressurizing the driver section with helium until the membrane ruptured. Sham-exposed rats were subjected to the same procedures except shockwave overpressure. Following exposure, rats were observed until return of righting reflex, returned to home cage, and observed periodically for 6 hours. Experimenters were blind to group assignment (sham or bTBI).

### Injury Assessment

The time taken to regain righting reflex following isoflurane termination was used as a metric for initial recovery from blast injury. At post-injury day 9, a composite neuroscore motor assessment was used to look for post-traumatic gross neurological dysfunction. This test assessed nine neurologically-related responses. Scores between 0 (severely impaired) and 2 (normal) were used for the first six tasks: left and right forelimb flexion during tail suspension, left and right hind limb flexion with forelimbs flat on surface and hind limbs elevated by tail suspension, and resistance to lateral pulsion from the left and right. Additionally, an inclined plane was used to test the rat’s ability to stabilize on an angled platform at three body positions: right side facing down the plane, left side facing down the plane, and rear end facing down the plane. A score was assigned to each body position depending on ability to stabilize: 0 (no stabilization); 1 (stabilization at 37.5°); 2 (stabilization at 40°); 3 (stabilization at 42.5°); 4 (stabilization at 45°). The composite score was the sum of all tests.

### Apparatus

Cognitive and drug self-administration experiments were conducted in an operant conditioning chamber (31.8 × 25.4 × 26.7 cm, Med Associates) enclosed in a sound-attenuating box. The right wall was equipped with two retractable levers on either side of a central food receptacle. A pellet dispenser delivered 45 mg sucrose tablets (cat#1811255, Test Diet) to the food receptacle. Chambers were also equipped with a house light and stimulus lights located above each lever. Commercially available software (MED-PC, Med Associates) was used for chamber programming and data collection.

### Experiment 1: Effects of bTBI on cognitive function

**Figure 1:**
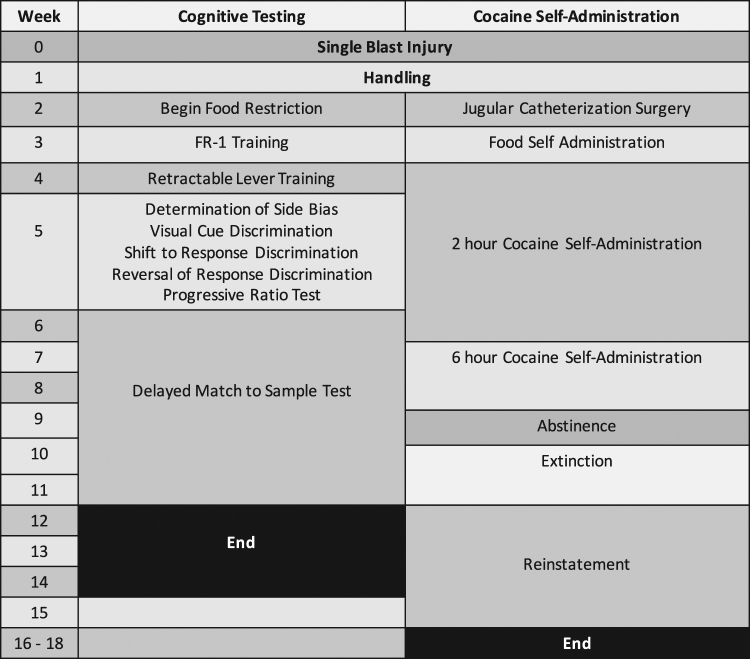
Timeline illustrating the sequence of behavioral tests in experiments one and two.

A total of 24 rats (n = 12 sham, n = 12 bTBI) underwent experiment 1. A timeline of experimental procedures is depicted in Fig. 1.

### Operant Set-Shifting and Reversal Experiments

The design of the task was adapted from^56,57^. Sessions were conducted daily. Rats began food restriction three days prior to the onset of the pre-training phase and at one day prior, rats were given 20 sucrose tablets in their home cage as exposure to the food reward.

#### Lever Press Training

The initial pre-training phase consisted of lever press training in which a single lever (left or right, counterbalanced across groups) was extended, and a press resulted in the delivery of a single reinforcer (sucrose tablet). After a criterion of at least 50 reinforcers in a given 30 min session was met, rats were then trained on the opposite lever using the same procedure.

#### Retractable Lever Training

The next pre-training phase consisted of sessions of 90 individual trials in which a single lever (either left or right, randomly selected without replacement within each pair of trials) was extended for a maximum of 10 sec. A lever press within this time period resulted in delivery of a single reinforcer, retraction of the lever, and a 10-sec time out period with the house light extinguished. Each new trial began with illumination of house light and extension of a lever. If a rat failed to press the lever within the 10 sec, the lever was retracted, an omission was counted, and the next trial began 10 sec later. Rats had a minimum of 4 sessions with this training and remained at this phase until a criterion of fewer than 15 omissions in a session was met.

#### Determination of Potential Side Bias

The end of the pre-training phase was marked with a single session to determine each rat’s potential side bias as described^58^. The session consisted of 7 individual trials with two phases to each trial. The first phase consisted of an illuminated house light and both levers extended. A lever press on either side resulted in delivery of a single reinforcer and retraction of levers. The second phase consisted of a “correct” and “incorrect” lever response. Both levers were again extended, but only a press on the lever opposite to the lever chosen in the first phase resulted in delivery of a reinforcer and beginning of new trial. This was a “correct” response. If the same lever from the first phase was chosen in the second phase (“incorrect”), the levers were retracted without reinforcer delivery, house light turned off, and then the second phase would be repeated until the rats made a “correct” response. The biased side was then determined by the greater number of lever presses in the first phase.

#### Visual-Cue Discrimination

The visual-cue discrimination training phase required the rat to identify the correct lever by following a visual cue light that was illuminated over the reinforced lever. Each session consisted of 20-sec trials in which both levers were extended and a single cue light was presented (left or right, randomly selected without replacement within each pair of trials). A correct lever press on the cue-presented side resulted in delivery of a single reinforcer, cue light turned off, and retraction of the levers. After a 10-sec time out, the next trial began. An incorrect lever press (side opposite the cue light) resulted in retraction of levers, cue light turned off, and no reinforcer delivery. If the rat failed to press a lever within 10 sec, an omission was recorded, the levers were retracted, and cue light extinguished. Rats were given the criterion of 10 consecutive correct trials (and at least 30 total trails, excluding omissions), with a maximum number of trials of 150 per session. If rats failed to meet criterion in a single session, they received additional sessions on subsequent days. Rats continued this task until they met criterion.

#### Shift-to-Response Discrimination

After meeting the criterion for the visual discrimination task, rats were tested the next day on a shift-to-response discrimination. The response (position) discrimination phase forced the rat to shift selection strategy from the visual-cue rule to a spatial rule in which the correct lever was independent of the cue light (e.g., always the left lever). A lever side was assigned to each rat opposite to their previously determined side bias. The session began with 20 “reminder” trials of the visual-cue rule then shifted to the new rule which was to ignore the cue and instead press only the right or left lever. These trials tested 24-hour recall of the visual discrimination and established performance on the visual-cue rule immediately prior to the shift^58^. Besides reward contingencies, trials were identical in presentation to those in the visual-cue discrimination phase. After the 20 “reminder” trials, the cue light continued to illuminate in randomized fashion but had no bearing on which lever was correct. Rats were given the criterion of 10 consecutive correct trials once the spatial rule was introduced and a maximum number of trials of 200 (including “reminder trials”) per session. If rats failed to meet criterion in a single session, they received additional sessions on subsequent days. Errors were classified as “perseverative”, “regressive”, or “never reinforced” if they occurred before meeting an intermediate acquisition criterion of 75% correct responses per block of 16 trials, after meeting this intermediate criterion, or if the incorrect response was not associated with the cue light^56^.

#### Response reversal test

Once rats met criterion, a response discrimination reversal test was given the following day. This test began with 20 “reminder” trails of the previously learned spatial rule then shifted to a new spatial rule in which the correct lever was on the opposite side. Rats were again given the criterion of 10 consecutive correct trials once the new spatial rule was introduced and a maximum number of trials of 200 (including “reminder trials”) per session. If rats failed to meet criterion in a single session, they received additional sessions on subsequent days until they met criterion.

#### Progressive Ratio

To determine the motivation to obtain the sucrose reinforcer, a progressive ratio test was conducted the day following response discrimination reversal. A single lever was extended (using the lever that was assigned to the rat in reversal test) for 1 hr in which rats freely pressed the lever on a progressive ratio schedule of reinforcement, where each ratio was calculated based on the formula described by^59^, using j = 0.18. The house light remained on for the entirety of the session, and no cue lights were used.

### Operant Delayed Matching to Sample (DMTS)

Immediately following the completion of the set-shifting experiment, working memory was tested using a DMTS experiment adapted from^60,61^. Delayed matching to sample was conducted in the same operant chambers with the same sucrose tablets as reinforcers. Because of the rat’s familiarity with pressing retractable levers, no initial lever press training was conducted. Each session consisted of 140 trials. A trial consists of three phases: sample, delay, and choice. A trial began with extension of single “sample” lever into the chamber (either left or right, randomly selected without replacement within each pair of trials). A lever press resulted in retraction of the lever and the delay period timer to start. During the delay phase, rats were required to nosepoke into the food receptacle to initiate the choice phase. This required rats to move away from the sample lever so they could not simply remain near the correct lever during the delay phase. Once the delay expired and a nosepoke was performed, both levers were extended giving the rat a choice of which lever to press. A response on the same lever as the sample phase (a correct choice) resulted in delivery of a single reinforcer, retraction of levers, and a 5 sec intertrial interval. A response on the opposite lever as the sample phase (an incorrect choice) resulted in retraction of levers, house light turned off, and a 5 sec time out before the next trial. In the trial following an incorrect response, a correction procedure was used to mitigate side bias. The correction procedure assigned the same lever as the previous trial instead of random assignment.

With initial learning sessions, no delay was used between sample and choice phases. Once the sample lever was pressed, rats could immediately initiate the choice phase by nosepoking into the food receptacle. Once the criterion of 80% correct choices for two consecutive sessions was met, sessions with delay sets were introduced. In delay set sessions, trials were split into blocks of 7, in which the delay durations were randomly presented once within each block. Three delay set sessions were used, and rats were required to meet the criterion of 80% correct choices for two consecutive sessions to advance from delay set 1 to 2 and delay set 2 to 3. Delay set 1 consisted of 0, 1, 2, 3, 4, 5, and 6 sec delays. Delay set 2 consisted of 0, 1, 2, 4, 8, 12, and 16 sec delays. Delay set 3 consisted of 0, 2, 4, 8, 12, 18, and 24 sec delays. Rats were tested for 10 consecutive days on delay set 3 and data from the all sessions was used for analysis. Due to inability to meet criterion on the DMTS task, 3 rats were removed from this experiment leaving a total of 21 rats with reported data (n = 11 blast injury, n = 10 sham).

### Experiment 2: Effects of bTBI on cocaine self-administration and drug seeking

A separate group of rats (n = 12 sham, n = 18 bTBI) underwent experiment two. A timeline of experimental procedures is depicted in Fig. 1.

### Jugular Catheterization Surgery

Rats underwent jugular catheter surgery similar to that described^62–64^. Rats were anesthetized with isoflurane (3–5% induction, 1–3% maintenance) and were implanted with a silicone catheter (Silastic, ID: 0.51 mm, OD: 0.94 mm, Dow Corning, Auburn, MI) into the right jugular vein, which exited through the intrascapular region and was connected to a cannula assembly. The cannula assembly consisted of a 22 gauge stainless steel cannula (Plastics One, Roanoke, VA) mounted on a base made from dental acrylic and nylon mesh (similar to that described in^65^), which was implanted subcutaneously. Rimadyl (carprofen, 5 mg/kg s.c.) was administered immediately following surgery and 24 h later. Catheters were flushed five days per week with 0.2 ml of heparinized saline (30 units/ml) and cefazolin (100 mg/ml).

### Food self-administration

After ≥5 days recovery from surgery, rats acquired food self-administration without food restriction. Sessions were 30 min in duration and began with turning on the fan (houselight remained off), extension of the two levers, and non-contingent delivery of a 45-mg sucrose tablet. Rats underwent fixed ratio-1 (FR-1) sessions, where a single press on the active lever (side of active lever counterbalanced between animals) resulted in delivery of a 45-mg sucrose tablet and illumination of a cue lamp above the active lever. The cue lamp remained lit for 5 sec, and a timeout period lasted 5 additional sec (10 sec total timeout period). During timeout, responses were counted but had no consequence. Responses on the inactive lever had no consequence. FR-1 sessions were 30 min in length but terminated early if 64 infusions were earned. FR-1 sessions continued until criteria were met (three consecutive sessions of ≥50 reinforcers and ≥2:1 ratio of active to inactive lever presses, minimum 5 sessions).

### Cocaine self-administration, extinction, and reinstatement

Rats self-administered cocaine in the same conditions as food self-administration, with the exception that intravenous infusion of cocaine (0.5 mg/kg) was the reinforcer and sessions were three hours in length. The infusion volume and time varied to deliver the appropriate dose from a 3.75 mg/ml stock solution infused at 17.4 ul/sec (e.g., 300 g rat received a 40 ul infusion over 2.3 sec). Rats were allowed to self-administer in 15 daily 2-hour sessions, then 10 daily 6-hour sessions. Catheter patency was determined after completion of the self-administration sessions using Brevital (methohexital, 10 mg/kg, i.v., JHP Pharmaceuticals, Rochester, MI) and any rat not meeting criteria for patency (sedation within 10 sec) was removed from the study. Nine total rats (n = 3 sham, n = 6 bTBI) were removed from the study due to loss of catheter patency and data were not included in analysis. The remaining rats then underwent abstinence for 5–11 days before extinction tests. Extinction tests were conducted in the same conditions as self-administration with the exception that no infusions were delivered. Following acquisition of extinction criteria (≤20 active lever presses for two consecutive days), rats were tested for cocaine primed reinstatement^66^. Reinstatement took place in three separate tests, and rats were required to meet extinction criteria again before the next test. Cocaine doses (0, 5, 10 mg/kg) were given according to a Latin squares design.

### Statistical Analysis

Data were analyzed by two-tailed Student’s t test and ANOVA (repeated measures when appropriate) followed by Holm-Sidak multiple comparisons. D’Agonstino and Pearson normality tests were performed, and Mann-Whitney U tests were conducted when normality tests failed (in which case data are represented as box plots). Statistical tests were performed using Prism 6.0 (Graphpad) or SPSS 21.0 (for 3-way ANOVAs). Significance was set at p = 0.05.

### Data availability

The datasets generated and analyzed during the current study are available from the corresponding author on reasonable request.

## Results

### Experiment 1: Cognitive testing following bTBI

**Figure 2:**
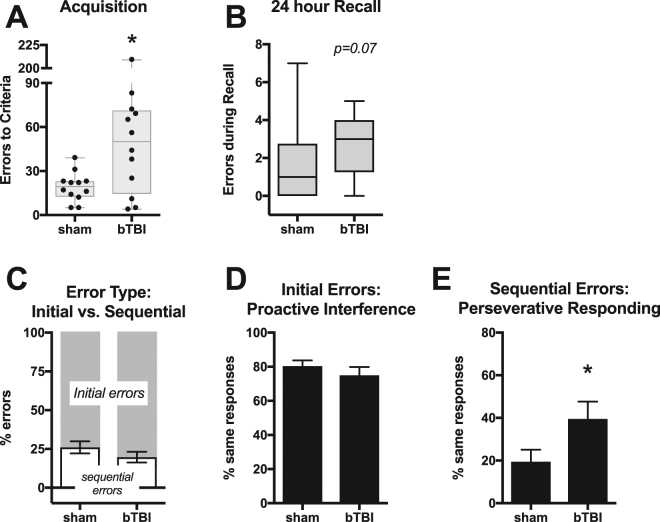
Acquisition and recall of the visual-cue discrimination task. (**A**) Number of errors committed during acquisition of task. (**B**) Number of errors committed in a 20-trial recall test performed 24 hours following acquisition criteria were met. (**C**) Proportion of errors during acquisition that were initial (not preceded by an incorrect trial) and sequential (preceded by an incorrect trial). (**D**) Proportion of initial errors that were committed on the same side as the previous trial. (**E**) Proportion of sequential errors during acquisition that were committed on the same side as the previous trial. Boxes represent median and quartiles, whiskers represent range, symbols are individual values. Bars represent mean ± SEM. N = 12/group. *p ≤ 0.05.

Following the bTBI procedure, there was no significant difference in recovery time between sham and bTBI rats (t(22) = 0.65, n.s.). Injury assessment with the composite neuroscore showed a trend for impairment nine days post-injury (t(22) = 1.8, p = 0.08), although our previous study using this found no significant effect of bTBI^44^. Despite this trend, no motor impairment was apparent in pre-training tests, which required numerous lever press responses each day of testing. Specifically, there was no difference between sham and bTBI rats in the number of days to meet criteria for FR-1 phase 1 (U = 68, n.s.), phase 2 (U = 72, n.s.), or retractable lever training (U = 66, n.s.). Conversely, there was a significant impairment in bTBI rats in acquisition of a visual discrimination rule. In the visual-cue discrimination task, there was a significant increase in the numbers of errors committed (U = 36, p < 0.05; Fig. 2A) and the number of trials required to reach criteria (U = 37, p < 0.05; data not shown). There was also a trend for bTBI rats to have impaired recall of the task 24 hours following acquisition (U = 41, p = 0.07; Fig. 2B). There were no significant differences between sham and bTBI rats in omissions (U = 66.5, n.s.; Fig. S1A), latency to lever press (t(22) = 0.1, n.s.; Fig. S1B), difference in lever bias (t(22) = 0.3, n.s.; Fig. S1C) during the visual-cue discrimination task, nor in motivation to obtain sucrose in the progressive ratio self-administration task (t(22) = 0.6, n.s.; Fig. S1D). These findings suggest that none of these potential confounds were responsible for the impairment in acquisition of the visual-cue discrimination task observed in the bTBI group.

To determine if there were qualitative differences in errors during acquisition of the visual discrimination task, errors were categorized into initial errors (those preceded by a correct trial) or sequential errors (those preceded by an incorrect trial). There was no significant effect of bTBI on the proportion of sequential errors relative to total errors (Fig. 2C), suggesting that errors committed by both groups were similarly distributed between these two categories. Initial and sequential errors were further analyzed by measuring the proportion which resulted from the same response as the previous trial. In the case of initial errors, responding on the same lever can reflect local proactive interference, a phenomenon where memory of the previously reinforced trial interferes with performance on the current trial^67,68^. In the case of sequential errors, responding on the same lever can reflect local choice perseveration, a failure to adapt response selection strategy following a previously unreinforced trial. Among initial errors, there was no difference in same lever responding between bTBI and sham rats (Fig. 2D). Among sequential errors, there was a significant increase in the proportion of same responses exhibited by bTBI rats (t(19) = 2.1, p = 0.05; Fig. 2E). This suggests that after an incorrect response, bTBI rats were more likely than shams to have another error due to the same response. Despite the deficits in acquisition of the visual-cue discrimination task, all bTBI rats ultimately met acquisition criteria. Combined, these data suggest that bTBI rats are not impaired in a simple instrumental task (i.e., in FR-1 and retractable lever training), but require additional training to acquire a task that requires flexible response selection based on location of a visual cue.

**Figure 3:**
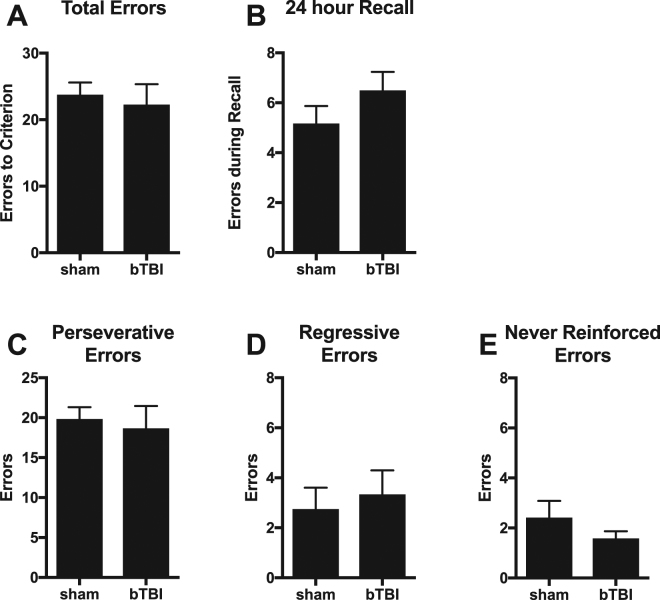
Performance in shift to response discrimination. (**A**) Number of errors committed during acquisition of set shift. (**B**) Number of errors committed in a 20-trial recall test performed 24 hours following acquisition criteria were met. (**C**–**E**) Analysis of error type during acquisition of set shift: (**C**) Perseverative errors, (**D**) Regressive errors, (**E**) Never reinforced errors. Bars represent mean ± SEM. N = 12/group.

**Figure 4:**
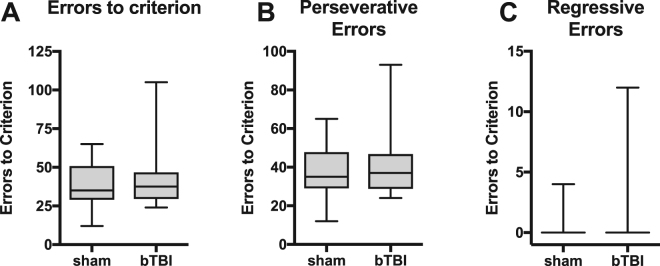
Performance in reversal of response discrimination. (**A**) Number of errors committed during acquisition of reversal. (**B**,**C**) Analysis of error type during acquisition of reversal: (**B**) Perseverative errors, (**C**) Regressive errors. Boxes represent median and quartiles, whiskers represent range. N = 12/group.

Next, rats were tested in a set-shift task. Acquisition of the set-shift task requires a shift from the visual discrimination rule to a location (response)-based rule^56^. We hypothesized that bTBI rats would be impaired in this task because inactivation of the mPFC results in set-shift deficits^56,69^, and we have previously characterized bTBI-associated changes in the mPFC that endure for at least 1 month^43^. Surprisingly, there was no difference between sham and bTBI rats in the errors committed during acquisition (Fig. 3A) or recall (Fig. 3B) of the set-shift task. We further analyzed errors into perseverative, regressive, or never reinforced (see Methods and^56^). These types of errors are thought to reflect suppression of the previously acquired strategy, maintenance of the newly acquired strategy, and acquisition of a new strategy, respectively^58,69^. There were also no differences in the number of any specific error types committed during acquisition (Fig. 3C–E). Thus, bTBI rats were able to shift their response strategy in a similar manner to sham rats. After acquisition of the set-shift, rats were tested in a reversal task. Response reversal is another measure of cognitive flexibility but requires reversal of the previous location rule as opposed to the extradimensional shift (shifting to a different type of rule) required for the set-shift. This reversal is impaired by disruption of transmission in orbitofrontal cortex, but not the mPFC^70,71^. Similar to the set-shift task, there were no differences between sham and bTBI rats in the number of errors to criteria or the number of perseverative or regressive errors in the reversal task (Fig. 4).

**Figure 5:**
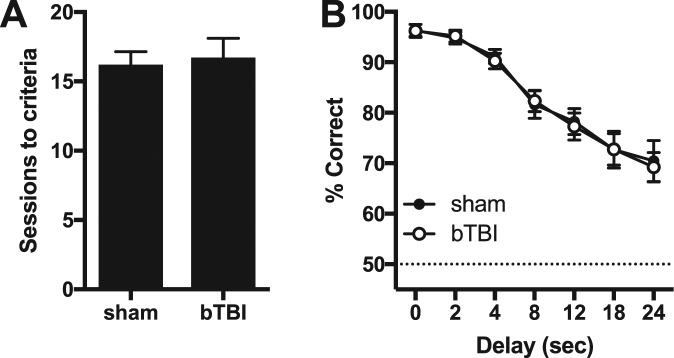
Acquisition and performance of the delayed matching to sample task. (**A**) Number of sessions required to meet criteria from the beginning of the task until completion of delay set 2. (**B**) Percentage of correct responses across delay periods. Symbols represent mean ± SEM. N = 12/group.

Problems with working memory have also been reported in mTBI patients^72^. To test for deficits in working memory in our bTBI model, rats were next tested in an operant DMTS task, an mPFC-dependent working memory task^73,74^. There was no difference in the number of sessions required to acquire the task (Fig. 5A), nor was there a main effect of bTBI or interaction with delay period in the DMTS task (Fig. 5B).

### Experiment 2: Effects of bTBI on cocaine self-administration and drug seeking

**Figure 6:**
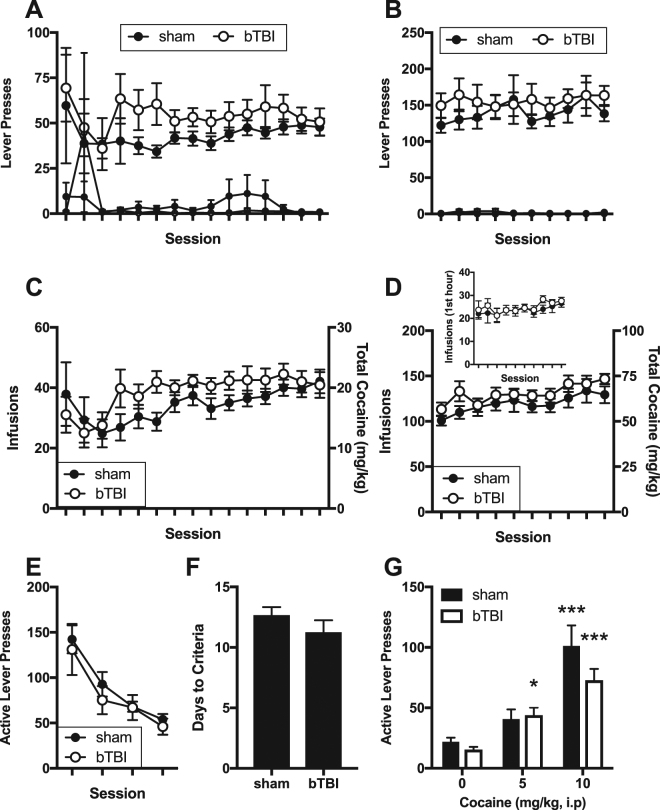
Cocaine self-administration and subsequent drug seeking. (**A**,**B**) Active (large symbols) and inactive (small symbols) lever responses during the (**A**) 15 days of 2-hour and (**B**) 10 days of 6-hour cocaine self-administration sessions. (**C**,**D**) Number of infusions earned and corresponding total cocaine intake during (**C**) 15 days of 2-hour and (**D**) 10 days of 6-hour cocaine self-administration sessions. Inset in panel D shows cocaine intake during the first hour of the 6-hour sessions. (**E**) Active lever responses during the initial four days of cued extinction sessions. (**F**) Number of days required to meet criteria for extinction prior to the first reinstatement test. (**G**) Active lever responses in cocaine primed reinstatement tests. Symbols represent mean ± SEM. N = 9 sham and 12 bTBI rats. *p ≤ 0.05 compared to 0 mg/kg cocaine in same group.

As in Experiment one, there were no significant differences in recovery time (t(27) = 0.95, n.s.) or composite neuroscore (t(27) = 0.74, n.s.) after injury. Unlike acquisition of the visual-cue discrimination task, there were no differences between sham and bTBI in acquisition of food self-administration (Fig. S2). There was also no main effect of bTBI or interaction with lever and/or sessions in 2- or 6-hour sessions when analyzing lever presses (Fig. 6A,C). Analysis of the number of infusions earned during these sessions also failed to find a significant effect of bTBI on drug intake or interaction with session (Fig. 6B,C). Extended access drug self-administration such as that in the 6-hour sessions can lead to an escalation of drug intake during the initial period of access^75,76^. To determine if bTBI altered escalation, we measured intake during the first hour of each 6-hour session (Fig. 6D inset). First hour responding was similar between groups throughout the sessions. Thus, bTBI had no significant effect on cocaine self-administration under short or extended access conditions. Based on the previous demonstration that our bTBI model leads to chronic changes in mPFC^43^ and extensive evidence that the mPFC regulates drug seeking^77,78^, we next measured extinction and reinstatement of drug seeking. There was no effect of bTBI on drug seeking in the initial four days of cued extinction sessions (Fig. 6E), nor on the number of days required to meet extinction criteria (Fig. 6F). In cocaine primed reinstatement, 5 mg/kg cocaine resulted in a significant increase in active lever presses in bTBI (p = 0.013, d = 0.94), but not sham rats (p = 0.11, d = 0.53). 10 mg/kg cocaine significantly reinstated drug seeking in both groups of rats (p < 0.001), but between group comparisons did not reveal significant differences between bTBI and sham rats at any dose. These data suggest that bTBI rats are not significantly more sensitive to the effects of cocaine to trigger drug seeking.

## Discussion

In this study, we found that a single exposure bTBI event led to impairment of learning that was dependent on experimental conditions (i.e., Experiment one vs. Experiment two). Despite the initial impairment in Experiment one, which was assessed 5 weeks after injury, there were no apparent differences in measures of cognitive flexibility. Once a visual discrimination rule was learned, bTBI rats successfully performed an extradimensional shift (ignore visual cue rule and acquire place rule) and reversal of the new rule (ignore old place and go to opposite place) within the same week. There was also no deficit in working memory as assessed by the operant DMTS task 11 weeks following injury. In contrast to Experiment one, bTBI had no effect on acquisition of food self-administration, which was assessed 3 weeks after injury. In cocaine self-administration, there was no difference between sham and bTBI rats in lever pressing or drug intake under short or extended access conditions 4–8 weeks after injury. When cocaine was unavailable, drug seeking (the first week of extinction responding) was similar between sham and bTBI groups 10 weeks following injury. When drug seeking was reinstated with priming injections of cocaine between 12 and 15 weeks after injury, bTBI rats significantly increased responding with the lower dose of cocaine, while sham rats did not. This difference was not confirmed by between-group measurements, suggesting that differences in the power to detect within-group effects (arising from minor differences in sample size, baseline responding, and variance) are responsible for the findings. Following higher dose of cocaine, both groups of rats had a similar magnitude of reinstatement, although there was a trend for bTBI rats to have lower responding than shams.

The finding that bTBI impaired learning in the visual-cue discrimination task (Experiment one) but not in the food self-administration task (Experiment two) is not surprising considering the differences in task requirements. In the visual-cue discrimination task, bTBI had an increased number of errors required to meet the acquire criterion. This task required flexible response selection based on location of a visual cue and had a stringent criterion for acquisition. In experiment two, bTBI had no significant effect on performance of FR-1 food self-administration. This simpler task involved static response selection guided by spatial and egocentric cues (e.g., the lever in the back-left quadrant was always correct) and had less stringent acquisition criteria. Further analysis of the visual-cue discrimination data did not reveal differences in the number of omissions, latency to lever press, or overt bias to one lever. Moreover, there was no significant difference between bTBI and sham rats in operant learning during the pre-training phase of operant set shifting or during food or cocaine self-administration. Thus, bTBI did not result in performance deficits due to general attentional deficits that could have reduced recognition of trial initiation or altered the latency to response, nor did it produce a non-specific bias in overall lever preference, impair operant conditioning, or reduce motivation to obtain food reward. The finding that bTBI impaired acquisition of a visual-cue discrimination task but did not affect set shift or reversal of this task is consistent with a previous report by Vonder Haar *et al*.^79^. In this study, frontal controlled cortical impact injury delayed re-acquisition of an operant-based auditory cue discrimination task, but reversal of the task was unimpaired^79^. Frontal cortical impact injury has also been demonstrated to impair re-acquisition of an olfactory cue discrimination, although reversal of the task was also impaired^80^. Thus, the finding of impaired cue discrimination has been reported across sensory modalities and tests with different task requirements, although response flexibility after discrimination learning may be specific to cue type and/or task requirements.

It is notable that despite significant group differences in the number of errors committed during visual-cue discrimination, several bTBI rats were in the range of shams (Fig. 2A). We have previously reported heterogeneity in alcohol drinking following the same injury, where median split analysis revealed that the upper 50% of bTBI rats drank significantly more than the upper 50% of shams^44^. In the current study, bTBI rats did not fit an apparent bimodal distribution, so median split was not performed. However, the heterogeneity in performance on the visual-cue discrimination task is reminiscent of that reported in clinical studies of cognitive recovery following mTBI^14–17,81^.

Analysis of error types revealed that bTBI rats had a significantly greater proportion of local perseverative responses in errors that were sequential in nature. Thus, bTBI rats more commonly failed to adapt their behavior in response to the negative feedback during the previous incorrect trial. However, there was no difference in perseverative errors observed during the set shift, and within the bTBI rats, perseverative errors committed during visual-cue discrimination was not correlated with perseverative errors committed during the set shift (F(1,8) = 0.001, n.s.). Following acquisition of the visual-cue discrimination, there was no difference in strategy set shifting between sham and bTBI rats. This was a surprising finding, as our previous work demonstrated differences in fractional anisotropy (FA) in the mPFC 30 days following bTBI^43^. We have previously found significant correlations between differences in FA within the hippocampus and motor cortex with performance in the Morris water maze, and FA within the frontal cortex has been shown to correlate with impairment in executive function following blast injury in humans^46^). Because set shift from visual cue to response has been demonstrated to be altered by mPFC infusion of bicuculline (GABA-A antagonist) or bupivacaine (voltage-gated sodium channel blocker)^56,69^, we hypothesized that bTBI rats would either commit more errors during acquisition of the shift-to-response discrimination, or that acquisition criteria would not be met.

Rats in Experiment one were also tested in an operant DMTS task to assess working memory function. bTBI had no effect on acquisition of this task and did not alter performance in delays up to 24 seconds. In contrast, delayed non-matching to sample and delayed alternation were disrupted ~30 days following mTBIs that used weight drop^82^ or controlled cortical impact^83^. An operant version of the delayed non-matching to sample was impaired for over 30 days in a cortical impact model, but the injury was severe enough to result in significant cavitation of the frontal cortex^84^. Our finding that DMTS was unaffected by bTBI is consistent with the lack of effect in the set-shifting task, as both of these tasks are disrupted by pharmacological inhibition of the mPFC^56,69,73,74^. It is possible that other factors underlie the lack of effect in DMTS. For example, rats were tested in visual cue discrimination, set-shifting, and reversal prior to DMTS. This prior training on relatively complex tasks could have altered performance on DMTS and masked potential group differences. DMTS was also conducted between 6–11 weeks following injury, and potential deficits may have resolved during this time.

We also found that cocaine self-administration did not differ between bTBI and sham treated rats. To our knowledge, this is the first study of intravenous drug self-administration in a preclinical model of TBI. In fact, despite evidence of increased substance abuse following TBI^5–9^, there are very few preclinical studies of addiction liability following TBI^85^. Mayeux *et al*. found that TBI increased alcohol intake in rats with a previous history of alcohol drinking^86^. Furthermore, the authors found that the amount of increase was associated with pre-injury intake, a phenomenon described in human injury^87–89^. In alcohol naïve mice, cortical impact TBI increased alcohol intake and conditioned reward in female mice that were injured early in life (P21), but not females that were injured at P60 or males that were injured at P21 or P60 when tested at P80~P100^90^. We previously reported a modest effect of bTBI increasing alcohol intake ~30 days post-injury in adult rats that were alcohol naïve pre-injury^44^. Thus, preclinical data suggests that TBI can increase subsequent alcohol intake, but age, sex, and prior alcohol history are all factors that can modify this relationship. There is also evidence that TBI can increase the conditioned rewarding effects of cocaine. In juvenile mice exposed to a cortical impact injury, conditioned place preference for cocaine was elevated when measured three weeks following injury^91^. It is unclear if the discrepancy between our findings and this study is due to age of injury, species difference, or known inconsistencies between conditioned place preference and self-administration studies^92–94^. In drug seeking tests following cocaine abstinence, we found no effect of bTBI on extinction responding or cocaine-primed reinstatement.

We found that acquisition of a learned discrimination following voluntary intake may be particularly sensitive to the effects of mTBI. Our bTBI model is a closed-head model of mTBI that does not result in significant changes in unconscious time or gross neurological function^43–45^, suggesting that it resembles very mild injury. This model was used previously to determine behavioral and brain structural changes following exposure to shockwave overpressures simulating mild blast TBI^43,45^, although no injury assessment was made in the current study. The parameters of blast used here are the same as those we used to characterize the model using MRI^43^, and the blast exposures are highly consistent (mean peak overpressure = 450.8 kPa, coefficient of variation = 11.2%). Rodent injuries produced using this model are solely attributable to head exposure to shockwave overpressure as the body is protected using a metal cylinder, head movements are minimized to limit head rotational accelerations well below the threshold for injury^43^, and rodents are placed off axis from the shock tube to avoid exposure to exhaust gases^43,95^. Whereas peak overpressure magnitudes from this model are somewhat greater than other models that incorporate shockwave exposure inside the shock tube^96–100^, peak shockwave overpressure is not the most appropriate injury metric. Shockwave duration also influences injury onset and severity and should be accounted for in assessments of shockwave exposure^101^. Accordingly, the area under the positive overpressure versus time curve, also known as impulse magnitude, is often used to assess shockwave exposure severity. As such, impulse magnitude in the current study (80 kPa*ms) is in line with other studies incorporating shockwave exposures inside the shock tube. For example, Perez-Garcia exposed rats to 74.5-kPa with 4.8-ms duration shockwaves inside the shock tube and reported an impulse magnitude of 175.8 kPa*ms^99^. That study characterized their exposure severities as ‘low-level’. Another recent study produced 207-kPa shockwaves with 2.5-ms durations inside the shock tube^96^. Assuming a Friedlander waveform shape, calculated impulse magnitude for those exposures would be 190 kPa*ms. Those exposures produced brain and lung edema, which were absent in the current model. Therefore, based on the severity of shockwave exposure in terms of impulse magnitude, a lack of significant pathological changes such as brain edema or hemorrhage, and modest behavioral changes demonstrated previously^43,45^, the present injury severity was graded as mild.

**Figure 7:**
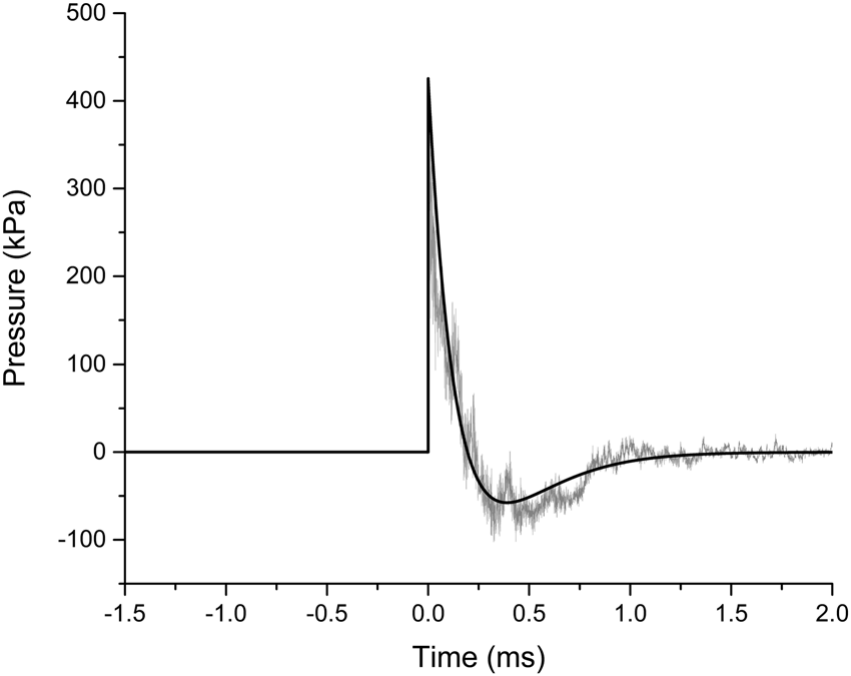
Characteristics of blast wave at the site of head placement. Recorded shockwave pulse is shown in gray and the theoretical Friedlander waveform is shown in black.

A possible limitation of this injury model includes the incorporation of shockwave exposure outside the shock tube, whereas a majority of recent studies have incorporated shockwave exposures with the animal positioned inside the tube. However, as indicated above, our focus on placement of the rat lateral to the shock tube axis to avoid exhaust gases from the shock tube partially minimizes this effect. Likewise, our fully-characterized shockwaves are consistent with Friedlander waveforms. Overpressure rise times are an average of 6 microseconds from ambient pressure to peak overpressure and our waveform represents the Friedlander waveform shape associated with a blast wave (Fig. 7). Therefore, this injury model, which has been well-accepted in the literature^43,45,95^, produces shockwave exposures representative of open field blast and consistent with previous studies from other groups.

Importantly, acquisition of a simple discrimination task (food self-administration in Experiment 2) was not impaired, supporting clinical findings that more advanced cognitive testing may reveal cognitive deficits in mTBI^17,18^. If changes in drug seeking are particularly vulnerable to injury, this could have important clinical implications for susceptibility to drug craving in individuals who sustain a brain injury. Further studies will be needed to determine if drug craving induced by other types of stimuli are also increased following injury and if environmental enrichment can improve the cognitive and addiction-related outcomes reported here as it has with other TBI-related outcomes^102–105^. Our findings also raise important questions regarding the generalizability to other drugs of abuse, effects of repeated injury, and effects on drug seeking if injury occurred during abstinence.

## Electronic supplementary material

Supplementary Figures
