# Supplementary material for: Effects of Mild Blast Traumatic Brain Injury on Cognitive- and Addiction-Related Behaviors
Source: Sci Rep. 2018 Jul 2;8:9941. doi: 10.1038/s41598-018-28062-0 (PMC6028456; doi:10.1038/s41598-018-28062-0)
Supplement: Supplementary file 1 — Supplementary Figures [file 41598_2018_28062_MOESM1_ESM.pdf]

Effects of Mild Blast Traumatic Brain Injury on Cognitive- and Addiction-Related Behaviors

Matthew J. Muelbl, BS[1,2], Megan L. Slaker, PhD[1,2], Alok S. Shah, MS[3,4], Natalie N. Nawarawong, BS[1,2], Clayton H. Gerndt, BS[1,2], Matthew D. Budde, PhD[3,4], Brian D. Stemper, PhD[3,4], and Christopher M. Olsen PhD[1,2*]

[1]Department of Pharmacology and Toxicology, Medical College of Wisconsin, 8701 Watertown Plank Rd., Milwaukee, WI 53226

[2]Neuroscience Research Center, Medical College of Wisconsin, 8701 Watertown Plank Rd., Milwaukee, WI 53226

[3]Department of Neurosurgery, Medical College of Wisconsin, 8701 Watertown Plank Rd., Milwaukee, WI 53226

[4]Clement J. Zablocki Veterans Affairs Medical Center, 5000 W National Ave, Milwaukee, WI 53295

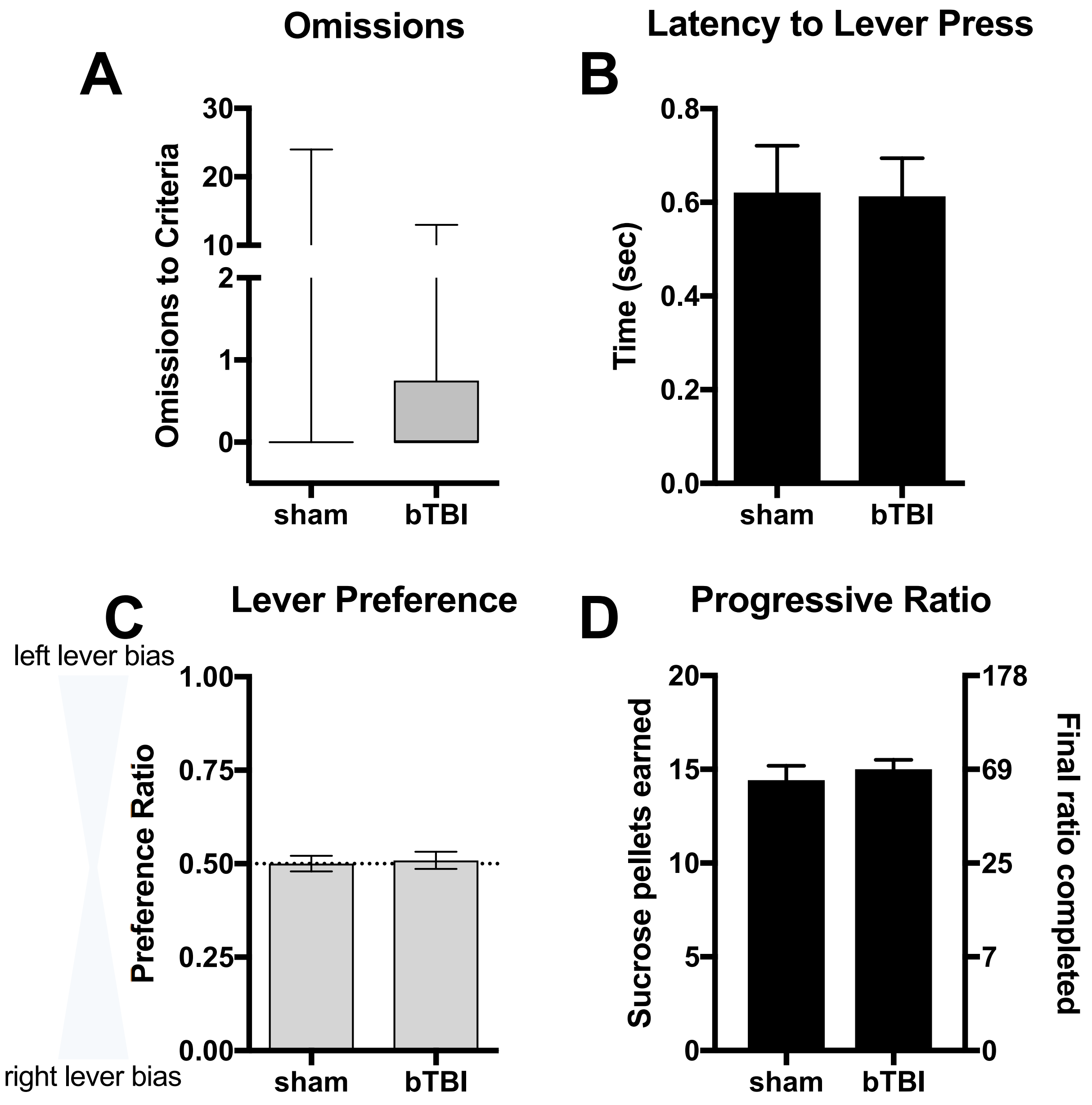

Figure S1: Additional metrics in the visual-cue discrimination task. A) Number of omissions. B) Latency to press lever. C) Overall lever preference was assessed by measuring the proportion of left and right lever presses across all trials (correct and incorrect). D) Sucrose pellets earned and final ratio completed in progressive ratio sucrose self-administration. Box represents median and quartiles, whiskers represent range. Bars represent mean±SEM. N=12/group.

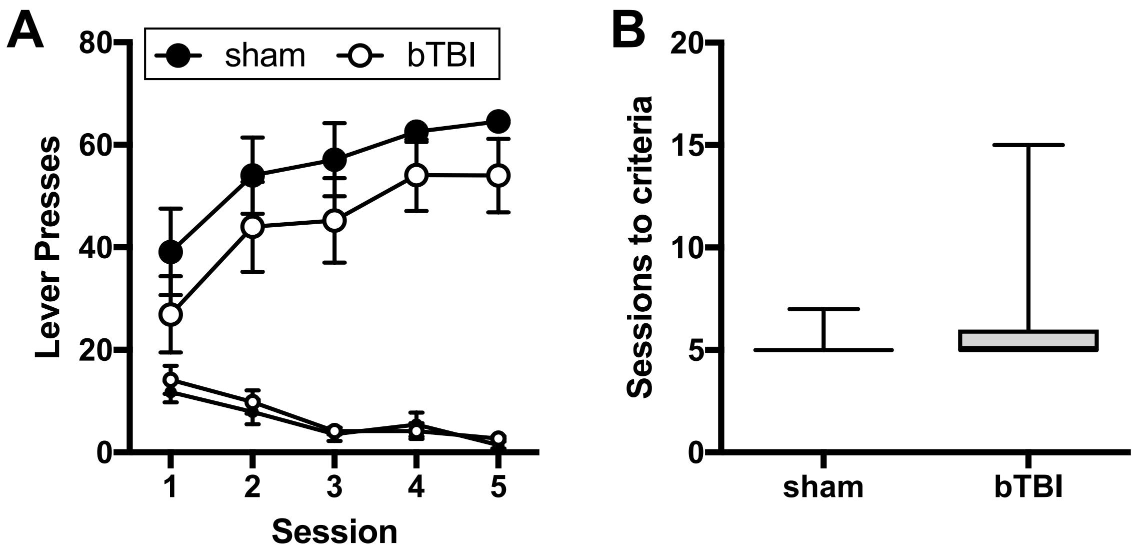

Figure S2: Acquisition of food self-administration. A) Active and inactive lever responses during the initial five days of food self-administration. B) Number of days required to meet criteria for acquisition of food self-administration. Bars represent mean±SEM. Box represents median and quartiles, whiskers represent range. N=9 sham and 12 bTBI rats.
